# Insight into the Relationship between the Causes of Off-Odour and Microorganism Communities in Xuanwei Ham

**DOI:** 10.3390/foods13050776

**Published:** 2024-03-01

**Authors:** Haoyi Wang, Xiaoyu Yin, Lu Zhang, Xuejiao Wang, Jiliang Zhang, Rongxin Wen, Jianxin Cao

**Affiliations:** 1 College of Food Science and Engineering, Kunming University of Science and Technology, Kunming 650500, China; zhisuix@163.com (H.W.); yinxiaoyu@kust.edu.cn (X.Y.); 15235243781@139.com (L.Z.); wangxuejiao173@hotmail.com (X.W.); zhangjl2348@163.com (J.Z.); 2 College of Life Sciences, Yantai University, Yantai 264005, China; wenrongxin810@163.com

**Keywords:** Xuanwei ham, microorganism communities, single molecule real-time sequencing, HS-SPME-GC-MS, correlation analysis

## Abstract

To expound on the correlation between the microorganism communities and the formation of off-odour in Xuanwei ham, the microorganism communities and volatile compounds were investigated in the biceps femoris (BF) and semimembranosus (SM) of Xuanwei ham with different quality grades (normal ham and spoiled ham). The single molecule real-time sequencing showed that differential bacteria and fungi were more varied in normal hams than in spoiled hams. Headspace solid-phase microextraction–gas chromatography (HS-SPME-GC-MS) results indicated that aldehydes and alcohols were significantly higher in spoiled hams than those in normal hams (*p* < 0.05). The off-odour of spoiled hams was dominated by ichthyic, malodourous, sweaty, putrid, sour, and unpleasant odours produced by compounds such as trimethylamine (SM: 13.05 μg/kg), hexanal (BF: 206.46 μg/kg), octanal (BF: 59.52 μg/kg), methanethiol (SM: 12.85 μg/kg), and valeric acid (BF: 15.08 μg/kg), which are positively correlated with *Bacillus cereus*, *Bacillus subtilis*, *Bacillus licheniformis*, *Pseudomonas* sp., *Aspergillus ruber*, and *Moraxella osloensis*. Furthermore, the physicochemical property and quality characteristics results showed that high moisture (BF: 56.32 g/100 g), pH (BF: 6.63), thiobarbituric acid reactive substances (TBARS) (SM: 1.98 MDA/kg), and low NaCl content (SM: 6.31%) were also responsible for the spoilage of hams with off-odour. This study provided a deep insight into the off-odour of Xuanwei ham from the perspective of microorganism communities and a theoretical basis for improving the flavour and overall quality of Xuanwei hams.

## 1. Introduction

Xuanwei ham is one of the three well-known dry-cured hams in China and has gained much popularity among consumers due to its unique flavour, rich nutrition, and delicious taste [1]. Recently, researchers have conducted in-depth studies on the functionality and absorption properties of bioactive peptides in Xuanwei ham, and it has been found that some of these peptides exhibit anti-hypertensive, antioxidant, and anti-inflammatory activities [2,3]. Xuanwei ham is produced in the Xuanwei district of Yunnan province, where the distinctive climate attributes and intricate geographical features provide an optimal natural setting for its processes of salting, drying, fermentation, and ripening, promoting the formation of the unique flavour of Xuanwei ham [4]. Salting and ripening are the crucial stages in the manufacturing of Xuanwei ham. The prolonged salting and ripening affect the biochemical and textural changes in muscles, albeit with varying degrees of intensity across different muscles [5]. The biceps femoris (BF) and semimembranosus (SM) muscles exhibit differential responses under the given circumstances [6]. The SM, as an external muscle, experiences accelerated dehydration and salt absorption during the initial phases of processing [6]. The biceps femoris (BF), as an intramuscular muscle, has a greater water content throughout its maturation, while the infiltration of salt into this muscle occurs at a comparatively advanced stage, specifically after a period of three months [6]. As a result, the proteolytic activity in BF is more pronounced compared to SM, leading to notable impacts on the textural profile and flavour profile [7]. 

At present, the processing method employed for Xuanwei ham is the traditional, workshop-style processing process, which is susceptible to the influence of bad weather, severely detracting from the quality of the ham. During the processing, the biochemical and microbiological activities affect the quality of the ham [8]. Different quality grades of ham may be produced due to the processing susceptibility of raw hams, even under consistent raw material and processing conditions [9]. The determination of the commercial grade of Xuanwei ham typically involves the utilisation of a traditional evaluation method known as the “three sticks method” [10]. This method entails inserting bamboo sticks into specific areas of the ham samples, namely the hip joint, knee joint, and sacral vertebra. Subsequently, professional technicians assess and score the odour attributes absorbed by these bamboo sticks [10]. The classification of the quality grade of the ham is based on the evaluation of changes in odour characteristics and intensities [11]. Proteolytic reactions and lipid oxidation are key processes in developing the characteristic flavour and quality grades of dry-cured ham [12].

The ham is typically subjected to an uncontrolled environment during processing, wherein numerous microorganisms are involved, notably bacteria and fungi originating from the raw materials and the surrounding surroundings [13]. A variety of microorganisms are present both inside and on the surface of the dry-cured ham, which is considered to play a crucial role in flavour development by facilitating proteolytic reactions and lipid oxidation during the product processing procedure [14]. Under specific circumstances, certain types of microorganisms undergo rapid proliferation inside or on the surface of the ham, resulting in the deterioration of the ham [15]. The presence of an unpleasant odour, along with accompanying deviations in colour and texture, renders the ham in question unsuitable for consumption [16]. *Bacillus* are ubiquitous and diverse in terrestrial and marine ecosystems, which facilitates their contamination of a variety of foods at different stages of the food chain [17]. The genus *Bacillus* includes species such as *Bacillus cereus*, *Bacillus licheniformis*, and *Bacillus subtilis*. For more than 40 years, *Bacillus cereus* has been recognised as an agent of food poisoning and has been associated with foodborne emetic and diarrheal syndromes [18]. *Bacillus licheniformis* has been associated with a variety of clinical syndromes and food poisoning events in humans, bovine toxaemia, abortions, and food spoilage events [19]. *Bacillus subtilis* is not usually considered a human pathogen but may occasionally contaminate food and cause food poisoning [20]. Total aerobic bacteria were higher in spoiled hams than in normal hams in the study of García et al. [21]. *Pseudomonas* sp. and *Moraxella osloensis* are both aerobic bacteria often associated with spoiled meat [22,23]. The corrosion of the hams is related to the proteolytic and lipolytic reactions in the ham, especially in which the odour generated is closely associated with the metabolic action of the microorganisms [10]. Several studies have investigated the compositions and dynamics of microbial communities, as well as their impact on the production of volatile compounds in dry-cured ham [10,15,24,25]. However, there is still a lack of comprehensive knowledge on the microbial diversity and flavour development of Xuanwei ham, especially the relationship between abnormal microbial growth and off-odour formation. Furthermore, no study comprehensively highlights the chemical, sensory, and volatile profiles of Xuanwei ham on the same experimental batch and comparatively on different muscles.

Recently, headspace solid-phase microextraction-gas chromatography-mass spectrometry (HS-SPME-GC-MS) has been widely used in the analysis of volatile compounds in meat products, offering a precise method for both qualitative and quantitative analysis of volatile compounds [26]. Due to the complex composition of meat products, analytes need to be extracted before introduction into the chromatographic system. Solid-phase microextraction (SPME) is a potential food analysis sample preparation method due to its simplicity, speed, solvent-free nature, analyte separation, and preconcentration [27]. HS-SPME-GC-MS has been widely used in the investigation of meat products, including the analysis of volatile profiles in fermented sausages and dry-cured hams [28,29]. 

Therefore, the aim of this study was to investigate the composition and diversity of microorganism communities and volatile compounds in the BF and SM muscles of Xuanwei ham with different quality grades (normal ham and spoiled ham). Meanwhile, comparisons of the physicochemical properties and quality characteristics of hams were also conducted. Furthermore, the potential correlations between core microorganisms and major volatile compounds were explored, and the core bacteria responsible for the off-odour of the Xuanwei ham were determined. This study may be helpful for regulating the spoilage in the production of Xuanwei hams and improving the flavour and quality consistency. 

## 2. Materials and Methods

### 2.1. Chemicals

The n-alkanes (C_5_ –C_25_) and gas chromatographic standard 1,2-dichlorobenzene were purchased from ANPEL Laboratory Technologies (Shanghai) Inc. (Shanghai, China). Other analytical grade reagents were obtained from Yunnan Sao Paulo Biotechnology Co., Ltd. (Xuanwei, China).

### 2.2. The Sampling of Xuanwei Ham

Xuanwei hams were manufactured according to Hu et al.’s processing procedures [30] with minor modifications. Briefly, the raw hind legs of pork were first trimmed and cut to remove blood stains and dirt. Then, they were salted in four stages. After each salting, the legs were classified and piled up. After a period of two months, the surface salt was washed off and dried until no water remained. The fermentation and ripening process lasted for 12 months. All of these procedures were conducted at D&H (Co., Ltd., Xuanwei City, China). The ripened Xuanwei hams were classified into four grades using the “three sticks” method, which involved assessing the intensity of the odour by smelling bamboo sticks. The hams that exhibited an intense meaty aroma in all three designated areas were classified as first-grade hams. The hams that exhibited an intense meaty aroma in two of the designated areas and in the remaining areas that did not exhibit any off-odours were classified as second-grade hams (normal hams). The hams that exhibited an intense meaty aroma in one of the designated areas and the other two remaining areas that did not exhibit any off-odours were classified as third-grade hams. Finally, the fourth-grade hams, referred to as spoiled hams, were identified by the presence of an intense off-odour in one of the three designated areas. Six hams were selected in a random manner from normal and spoiled hams, respectively. All hams were produced from the same batch under the same external conditions and processes. The outermost layers (approximately 0.5 cm) of Xuanwei hams were removed. Subsequently, the BF and SM muscles were quickly separated for further analysis. 

### 2.3. Determination of pH, Moisture Content, Water Activity (a_w_), and NaCl Content

The muscles were homogenised in a blender to measure the pH, moisture, aw, and NaCl content, respectively. The pH was measured with a pH metre (PHS-25, Shanghai INEIDIAN Scientific Instrument Co., Ltd., Shanghai, China). The moisture content was determined according to the method of Marusic et al. [31]. The *a*_w_ was measured using a LabMaster water activity metre (LabMaster, Novasina, Switzerland) according to the method of Zhu et al. [15]. The NaCl content was determined using atomic absorption spectrometry (AAS, Agilent Technologies, Santa Clara, CA, USA) according to the method of Gamela et al. [32]. 

### 2.4. Determination of Texture Profile

The texture profile analysis (TPA) was carried out using a texture analyser (TA-XT. Plus, Stable Micro Systems Ltd., Godalming, UK) according to the method of Liao et al. [33] with some modifications. The BF and SM muscles were cut into pieces (10 mm × 10 mm × 10 mm). The TPA analysis was performed under the following measurement parameters: P50 probe (50 mm diameter), pre-test speed 1.0 mm/s, test speed 1.0 mm/s, post-test speed 5.0 mm/s, trigger force 30.0 g, and strain 70%. 

### 2.5. Determination of Colour

The colour was measured using a Hunterlab Agera colourimeter (Hunter Associates Laboratory Inc., Reston, VA, USA) according to the method of Marusic et al. [31]. The values expressed as L*-value (lightness), a*-value (redness), and b*-value (yellowness) were obtained from three different cut areas of each muscle. 

### 2.6. Sensory Evaluation

The sensory analysis was performed according to the description of Hersleth et al. [34], with some modifications. Xuanwei ham was evaluated for red colour, lightness, hardness, meaty, unpleasant odour, and overall acceptance. Before the sensory evaluation, twenty panellists (10 males and 10 females, 21–25 years old) were chosen from a group of teachers, staff, and graduate students who have participated in professional sensory training courses. Sensory attributes were evaluated using a 10-point intensity line scale: red colour, 1 = pale and 10 = dark red; lightness, 1 = dull and 10 = bright; hardness, 1 = hard or loose and 10 = moderate toughness; meaty odour, 1 = nondetectable and 10 = intense; unpleasant odour, 1 = nondetectable and 10 = intense; overall acceptability, 1 = unacceptable and 10 = very favourite. Ham slices (1-mm thick) were served randomly on white plastic plates, labelled with randomised 3-digit numbers, and evaluated in a sensory chamber at 20–22 °C. Between successive ham samples, the panellists were given approximately 50 mL of water. The panellists were asked to indicate the appropriate scale point based on the intensity of their perception of each attribute. 

### 2.7. Determination of Electronic-Nose

Electronic-nose (E-nose) analysis was performed using a PEN3 E-nose (Airsense Analytics GmbH, Schwerin, Germany) according to the method of Yao et al. [35]. The PEN3 system contains 10 metal oxide gas sensors (W1C, W5S, W3C, W6S, W5C, W1S, W1W, W2S, W2W, and W3S) that detect olfactory cross-sensitivity information [36]. The response characteristic of each sensor was shown as follows: W1C (aromatic), W5S (broad range), W3C (aromatic), W6S (hydrogen), W5C (arom-aliph), W1S (broad-methane), W1W (sulphur-organic), W2S (broad-alcohol), W2W (sulph-chlor), and W3S (methane-aliph). 3.00 g of minced ham was placed into a 20 mL headspace vial and equilibrated in a water bath at 45 °C for 40 min. The sensor array was cleaned using treated pure air as a carrier gas to zero the signal response. The parameters for the sensors were set at an injection flow rate of 200 mL/min and an analysis time of 100 s. 

### 2.8. Determination of Thiobarbituric Acid Reactive Substances

Lipid oxidation was assessed by thiobarbituric acid reactive substances (TBARS) as described by Wang et al. [37] with minor modifications. TBARS values, expressed as mg/kg muscle of malondialdehyde, were calculated using the following equation:

TBARS (mg/kg) = (A_532_/Ws) × 9.48

where A532 was the absorbance (532 nm) of the assay solution, Ws was the muscle weight (g), and “9.48” was a constant derived from the dilution factor and the molar extinction coefficient [152,000 (mol/L)^−1^ cm^−1^] of the TBA reaction product. 

### 2.9. Determination of Volatile Compounds

Volatile compounds in Xuanwei hams were determined using headspace-solid phase microextraction (HS-SPME) combined with gas chromatography-mass spectrometry (GCMS-QP 2020 NX, Shimadzu Co., Kyoto, Japan). The minced ham (3.00 g) was put into a sealed headspace vial (VFAP-606050F-18M-100, CNW Technologies, Shanghai, China) with added 1 μL 1,2-dichlorobenzene as the internal standard. Subsequently, the 50/30-μm-thick divinylbenzene/carboxen/polydimethylsiloxane (DVB/CAR/PDMS) fibre (57328-U, Supelco Inc., Bellefonte, PA, USA) was exposed to the headspace of a headspace vial for extraction at 45 °C for 40 min. Finally, the volatile compounds absorbed were desorbed in the GC injector at 240 °C for 5 min, identified, and quantified using the GC-MS system equipped with a DB-WAX (30 m × 0.25 mm × 0.25 μm, Agilent Technologies, Santa Clara, CA, USA). The GC oven temperature programme was as follows: first increase, 40 °C for 3 min, 3 °C/min to 90 °C for 5 min; second increase, 3 °C/min to 200 °C; and third increase, 15 °C/min to 230 °C for 5 min; split ratio of 5:1. The flow rate of the high-purified helium as the carrier gas was 2.0 mL/min. The mass spectrum parameters were electron ionisation mode at 70 eV and a scanning range of *m*/*z* 35–500. The MS ion source and quadrupole temperatures were 200 °C and 230 °C, respectively. 

Volatile compounds were identified by comparing the mass spectra with reference mass spectra from the NIST21 library data with a similarity of 90%. For further confirmation, the retention index (RI) was calculated using a mixture of n-alkanes (C_5_–C_25_). Finally, the odour activity value (OAV) of the volatile compounds was calculated according to Chen et al.’s method [38]. 

### 2.10. Determination of Microbial Diversity

Total genomic DNA from the Xuanwei ham was extracted using the CTAB/SDS method [39] and monitored using 1% agarose gels. For bacteria, 16S rRNA, the front primer was using 27F(5′-AGAGTTTGATCMTGGCTCAG-3′) and the reverse primer was used 1492R(5′-ACCTTGTTACGACTT-3′). For fungi, ITS rRNA was amplified. The front primer, ITS1F(5′-CTTGGTCATTTAGAGGAAGTAA-3′), and the reverse primer, ITS2 (5′-GCTGCGTTCTTCATCGATGC-3′) were used. PCR products were detected on a 2% agarose gel and further purified using the QIAquick@ Gel Extraction Kit (QIAGEN, Hilden, Germany). Sequencing libraries were prepared for the 16S rRNA gene and the ITS rRNA gene using the SMRTbellTM Template Preparation Kit (PacBio, Menlo Park, CA, USA). Both libraries were sequenced on the PacBio Sequel platform, and the raw sequences obtained were multiplexed and quality-filtered through the PacBio SMRT portal. Potential chimeras were removed using the UCHIME algorithm, resulting in clean sequences. These sequences were then assigned to operational taxonomic units (OTUs) using UPARSE with a 97% identity threshold. The classification of each representative sequence was assigned using the SSUrRNA database from the Silva Database. 

### 2.11. Statistical Analysis

All experiments were independently carried out at least three times, and the results were expressed as mean ± standard deviation (SD). Statistical analyses were carried out with SPSS Statistics 25.0 software (IBM, Chicago, IL, USA). The data were shown as mean ± standard deviation. All the data obtained were submitted to analysis of variances (ANOVA), and the means were compared using the Tukey test (*p* < 0.05). Radar plots, principal component analysis (PCA) plots, histograms, and clustered heat maps were plotted using Origin2021. LEfSe, Wayne plots, and bimatrix clustered correlation heatmaps were plotted using the OmicStudio tool at https://www.omicstudio.cn/tool/ (accessed on 8 November 2023). 

## 3. Results and Discussion

### 3.1. Sensory Evaluation of Xuanwei Ham

**Figure 1:**
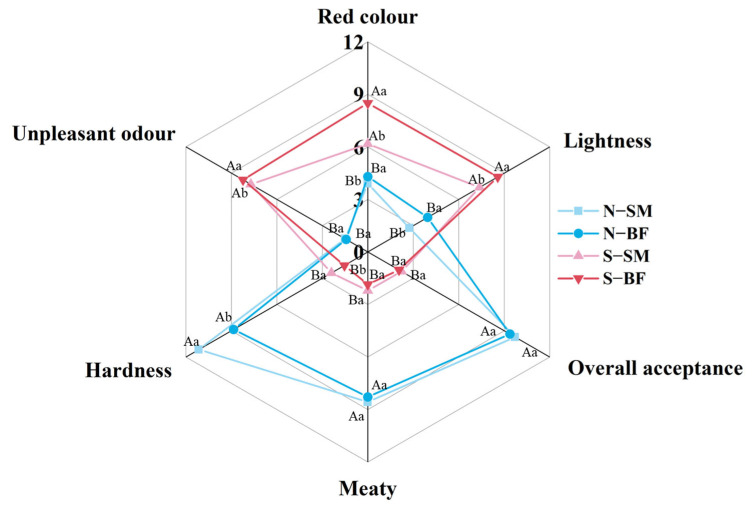
Sensory evaluation of different muscles in normal and spoiled hams. Different uppercase letters (A,B) indicate significant differences between hams with different quality grades for the same muscle (*p* < 0.05), and different lowercase letters (a,b) indicate significant differences between different muscles for the ham with the same quality grade (*p* < 0.05). N–SM: normal ham–semimembranosus; N–BF: normal ham–biceps femoris; S–SM: spoiled ham–semimembranosus; S–BF: spoiled ham–biceps femoris.

Radar charts of sensory evaluation (red colour, lightness, meaty, hardness, unpleasant odour, and overall acceptance) for the SM and BF of both normal and spoiled hams are shown in Figure 1. The overall acceptance of normal hams (N−BF and N−SM) was significantly higher than that of spoiled hams (S−BF and S−SM) (*p* < 0.05). Within the same muscle, the red colour, lightness, and unpleasant odour of spoiled hams (S−BF and S−SM) were significantly higher than those of normal hams (N−BF and N−SM) (*p* < 0.05), while the meaty and hardness scores of spoiled hams were lower (*p* < 0.05). This may be due to the fact that some unpleasant and pungent odours were larger and masked the aroma of the ham [40]. Within the same quality grade of ham, red colour and lightness scores were found to be significantly higher (*p* < 0.05), and hardness scores were significantly lower (*p* < 0.05) in BF than in SM, which may be due to the different biochemical reactions occurring at the two muscles as well as different microbial activity [5]. However, no significant difference was found between the SM and BF in terms of meaty and overall acceptance (*p* > 0.05).

### 3.2. Moisture, a_w_, pH, and NaCl Content Analysis

The physicochemical properties of the SM and BF muscles of both normal and spoiled hams are shown in Table 1. It was found that spoiled hams had significantly higher (*p* < 0.05) moisture contents (49.98–56.32 g/100g) than normal hams (39.44–46.88 g/100 g) in the same muscle. This result was consistent with the finding of Blanco et al. [41], who found that the moisture content was significantly higher in Spanish hams with bone taint than in normal Spanish hams, indicating that higher moisture content may be one of the causes of ham spoilage. The moisture content of BF was significantly higher than that of SM in hams of the same quality grade (*p* < 0.05), which could be due to their different parts in dry-cured ham [31]. The SM is located on the surface and is more susceptible to environmental influences, while the BF is wrapped by the SM and subcutaneous fat, rendering it less susceptible to environmental factors [31]. Consequently, the BF had a lower water loss compared to the SM.

*a*_w_ is the state in which water exists in the system, representing the degree to which it is bound (or degree of freedom) [15]. The higher the *a*_w_, the lower the degree of binding [15]. In this study, the *a*_w_ of spoiled hams ranged from 0.87 to 0.88, while that of normal hams ranged from 0.74 to 0.75. This indicates that the degree of binding of spoiled hams is lower than that of normal hams. Previous research has shown that when *a*_w_ > 0.83, dry-cured hams are more susceptible to microbial contamination, which can lead to ham spoilage [42]. Therefore, controlling *a*_w_ during the processing of ham can effectively inhibit the growth of most microorganisms [15]. Wu et al. [43] demonstrated that *a*_w_ decreased with the increasing ripening time of the Laowo ham. The *a*_w_ of Laowo ham reached 0.74 after undergoing a 9-month maturation period, which exhibited similarity to the results of normal Xuanwei ham ripened for one year in this study.

The pH of the spoiled hams ranged from 6.45 to 6.64, whereas the pH of the normal hams ranged from 5.74 to 5.79. These results are similar to the findings of Zhu et al. [15] in Mianning hams, who found the pH of normal ham was approximately 5.93, whereas that of deeply spoiled ham was around 6.51. According to Bosse et al. [44], microorganisms were more suitable for colonisation on dry-cured hams when pH > 6.0, indicating that spoiled hams were more susceptible to microbial influences.

The salting process of Xuanwei ham involved four stages: first at 4%, then at 3%, then at 2.0% and finally at 1.0%. Conducting salting in multiple stages facilitated salt penetration, while the subsequent drying process caused significant water loss from the ham, leading to an increase in salt concentration [31]. The NaCl content in normal hams was significantly higher (*p* < 0.05) compared to that in spoiled hams in the same muscle, which may be related to salt penetration during the curing and the rate of air drying [31]. The lower the salt content, the greater the water retention capacity of the ham, resulting in the higher moisture content observed in spoiled hams. Additionally, Lin et al. [45] found that a low salt content could result in an increase in pH, which was consistent with the pH results. For hams with the same quality grade, the salt content of the BF was significantly higher than that of the SM (*p* < 0.05). This finding aligns with the study by Ruiz-Ramírez et al. [46], who found that the SM was subjected to a higher salt concentration during salting, underwent more dehydration during the curing process, and exhibited a higher salt content compared to the BF muscle after drying. The salt concentration remained constant across the entire surface of the ham during the initial production process [31]. Due to the presence of a concentration gradient, the salt diffused from the SM (with a higher content) to the BF, leading to the BF, which contained more water, also exhibiting a higher salt concentration [31].

### 3.3. Lipid Oxidation Analysis

To examine the quality difference between spoiled and normal hams, we analysed the degree of lipid oxidation (Table 1). Lipid oxidation may alter the colour, flavour, texture, and nutritional content of foods [47]. Within the same muscle, the spoiled hams exhibited significantly higher TBARS values compared to the normal hams (*p* < 0.05). Generally, a TBARS value below one is considered acceptable for lipid oxidation; a TBARS value above one suggests excessive oxidation, potentially leading to rancidity in meat products [48]. In this study, the low TBARS value in normal ham indicated a low degree of oxidation, which contributed to the unique aroma of Xuanwei ham. The high TBARS value of spoiled ham, indicating a higher degree of lipid oxidation, produced a strong rancid flavour, which may be one of the reasons for the off-flavour of spoiled hams [49]. Similar results were observed in the Jinhua hams [9] and Mianning hams [15], demonstrating significantly higher TBARS value in spoiled hams compared to those in normal hams. Generally, the development of lipid oxidation in dry-cured hams relies on a number of factors, such as the raw material composition and characteristics, the processing conditions and the amount and type of additives and ingredients added to the product [50]. In this study, the difference between the spoiled ham and the normal ham might have correlations with the materials and processing environment [51]. Within the same quality grade, the TBARS value of SM was significantly higher than that of BF (*p* < 0.05). This higher TBARS value in SM could stem from its surface location, increasing its exposure to oxygen and susceptibility to oxidation [52]. These results affirm TBARS as a crucial indicator for assessing the quality of both spoiled and normal hams [53].

### 3.4. Textural Profile Analysis

Texture plays a crucial role in evaluating the quality of hams. The correlation between protein hydrolysis and the texture of dry-cured hams has been established during the production process, whereby the curing and ripening stages have been recognised as the primary factors impacting protein hydrolysis and the resulting texture of dry-cured hams [54]. The results of hardness, springiness, cohesiveness, chewiness, and resilience for SM and BF of both normal and spoiled hams are shown in Table 2. In this study, the normal ham had significantly greater hardness than the spoiled ham in the same muscle (*p* < 0.05), which was inextricably linked to its low moisture content, pH, and high salt concentration. According to Ruiz-Ramirez et al. [55], dry-cured ham with a lower pH was harder than those with a higher pH at the same moisture content and harder as the moisture content decreased. It was also observed that the hardness of the ham significantly decreased when the salt concentration was reduced from 1.5% to 0.75% [56]. Within the same quality grade, the SM exhibited significantly higher hardness compared to the BF (*p* < 0.05), which may be attributed to the fact that the SM had a lower water content than the BF. This finding was consistent with the results of Slovenian and Serta [5,57] on different muscle types of dry-cured hams. Generally, the chewiness varied in line with hardness [58]. In this study, compared to the spoiled ham, the normal ham had higher chewiness and springiness in the same muscle (*p* < 0.05). However, the difference in springiness in different muscles was not significant (*p* > 0.05), which agreed with the results in Slovenian dry-cured ham [59].

### 3.5. Colour Analysis

The colour is one of the most important indicators for evaluating the quality of dry-cured ham and affects consumer acceptability. The main colour-developing substances in hams are myoglobin-like and haemoglobin and their derivatives [60]. The results of L*, a*, and b* of normal and spoiled Xuanwei hams are shown in Table 2. Within the same muscle, the L* value of normal hams was significantly lower than that of spoiled hams (*p* < 0.05), which was attributed to the increase in pigment concentration caused by the reduction of moisture content in dry-cured hams, finally leading to the decrease of L* values [61]. Within the same quality grade, the L* value of SM was significantly lower than that of BF muscle (*p* < 0.05). 

Within the same muscle, the a* value of normal ham was significantly lower than that of spoiled ham (*p* < 0.05). According to Jens et al. [62], who conducted a spectral analysis of pigmented substances in Parma ham, this difference may be attributed to the increase in fermentation and ripening time, which led to the production of a large number of pigments. However, these pigments are not nitroso-myoglobins; they may be some trivalent iron-containing ionic compounds that are oxidatively stable [15]. The a* values of BF were significantly higher than those of SM (*p* < 0.05) in hams of the same quality grade, indicating that BF exhibited a redder colour than SM. The a* values were related to the air-drying process of dry-cured hams [63], where SM was in the process of stronger surface dehydration than BF, and SM had a significantly lower moisture content than BF (*p* < 0.05), and this may explain the observed difference in colour between BF and SM.

The b* value is typically unstable during processing but gradually stabilises during the maturation stage [31]. The b* values in spoiled hams were significantly higher (*p* < 0.05) compared to normal hams within the same muscle. The increase in b* value may be attributed to the yellow pigmentation resulting from the reaction between the products of lipid oxidation and the amines in the phospholipid head group or the proteins [15]. The b* values in fresh pork patties also showed an increase with higher lipid oxidation, as observed by Chang et al. [64]. Within the same quality grade, BF had higher L*, a*, and b* values compared to SM, which is consistent with the results of sensory evaluations and previous studies by Gou et al. [65] and Cilla et al. [66].

### 3.6. E-Nose Analysis

**Figure 2:**
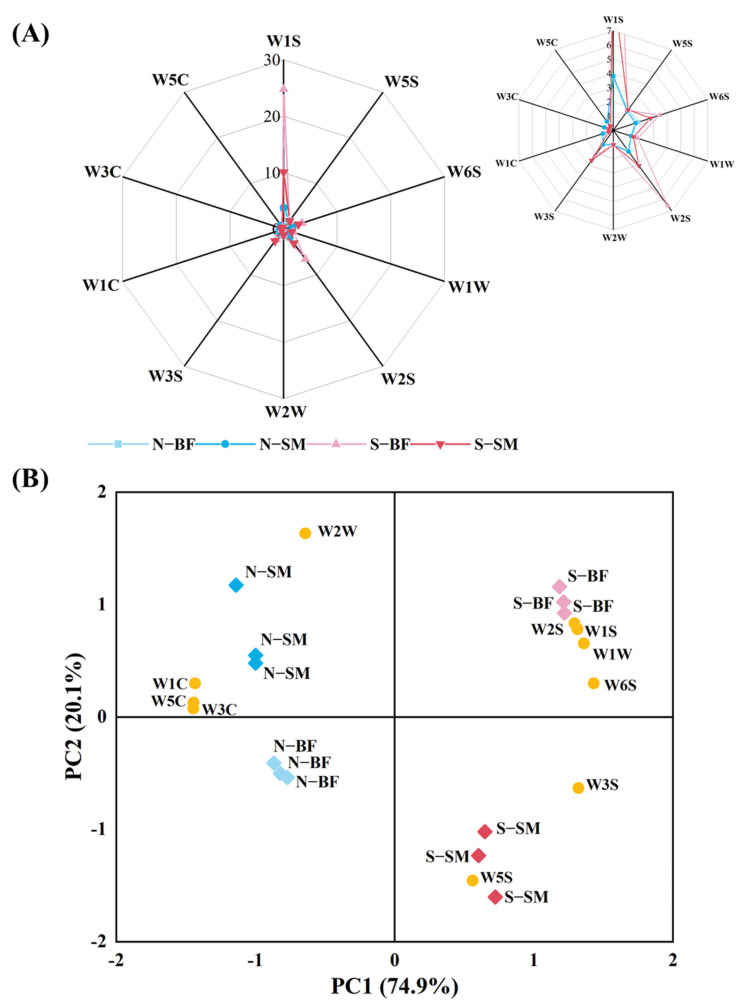
Radar plots of e−nose response data (**A**) and e-nose PCA scores (**B**) of different muscles in normal and spoiled hams. N−SM: normal ham−semimembranosus; N–BF: normal ham–biceps femoris; S–SM: spoiled ham–semimembranosus; S–BF: spoiled ham–biceps femoris.

The E-nose is sensitive to aromas in samples, and slight changes in volatile compounds may result in differences in sensor response [36]. As seen in Figure 2A, the W1S sensor exhibited the strongest response to volatile compounds in the spoiled ham. Furthermore, the W6S, W2S, and W3S sensors also showed strong responses to volatile compounds in spoiled hams, indicating a potentially higher concentration of methyl groups and alcohols in these samples. Conversely, the W1C, W3C, and W5C sensors demonstrated stronger responses to normal hams compared to spoiled hams, suggesting a higher presence of aromatic compounds in normal hams. The e−nose was employed to differentiate between normal and spoiled hams, as well as Xuanwei hams with different muscle types.

The PCA results are presented in Figure 2B. The first principal component (PC1) accounted for 74.9% of the sample variance and displayed a positive correlation with W5S, W6S, W1S, W1W, W2S, and W3S. The second principal component (PC2) explained 20.1% of the variance and exhibited a positive correlation with W3C, W6S, W5C, W1S, W1W, W2S, and W2W. The sample distribution demonstrated that PCA effectively differentiated the samples. Normal hams mainly occupied the second and third quadrants, positively correlating with the W1C, W3C, W5C, and W2W sensors, which indicated an abundance of aromatic, short-chain alkanes and sulphur-chlor compounds. Conversely, spoiled hams were predominantly located in the first and fourth quadrants and positively correlated with the W1W, W1S, W2S, W3S, W5S, and W6S sensors, indicating prevalent compounds such as methyls, sulphides, alcohols, aldehydes, and ketones. PC1 effectively discriminated between normal hams and spoiled hams, while PC2 distinguished between SM and BF. Consequently, the e-nose is an effective tool for distinguishing different quality grades of Xuanwei ham. However, identifying the specific compounds in these samples remained challenging.

### 3.7. Analysis of Volatile Compounds

#### 3.7.1. Volatile Compounds Content

The volatile compound contents in the SM and BF of normal and spoiled Xuanwei ham are shown in Table 3. A total of 89 volatile compounds were detected, including 21 alcohols, 19 esters, 15 aldehydes, 9 acids, 7 ketones, 3 alkanes, 4 pyrazines, 4 aromatics, 3 terpenes and 4 other compounds.

Alcohols are one of the most abundant volatile compounds in dry-cured ham [40], with a total of 21 alcohols identified. Straight-chain fatty alcohols are formed through lipid oxidation reactions, while methyl-branched alcohols may result from the strecker degradation of amino acids [67]. Due to their low threshold, alcohols are often considered crucial contributors to the characteristic flavour of dry-cured meat products [68]. In this study, higher levels of alcohol were found in spoiled hams, where the total alcohol content in the BF of normal hams was higher than that of SM (*p* < 0.05), consistent with a previous study on different muscles of Slovenian dry-cured hams [5]. Among the detected alcohols, ethanol exhibited the highest content (*p* < 0.05). Due to its relatively high odour threshold, ethanol has been deemed insignificant in the overall flavour contribution of Jinhua ham [69]. It is noteworthy that methanethiol, characterised by a sewage odour and rotten cabbage scent, was exclusively present in spoiled ham [70]. 

According to a previous report by Forss, D. [71], intramuscular fat in meat plays an important role in preserving flavour by acting as a solvent for flavour compounds within the muscle tissue and facilitating subsequent reactions. Esters primarily form through the esterification reactions of carboxylic acids and alcohols. Short-chain esters contribute to fruity flavours, whereas long-chain esters are associated with fatty flavours [72]. The content of esters is generally low in Spanish and French dry-cured hams but higher in Italian Parma ham and homemade ham [73]. This may be due to the fact that nitrates, which inhibit lipid oxidation, are absent in the processing of Parma ham but are added to Spanish and French hams [74]. In this study, Xuanwei ham exhibited a higher ester content, likely also due to the absence of nitrate usage [60]. Moreover, the ester content was higher in the spoiled ham compared to the normal ham (*p* < 0.05), consistent with a previous study on spoiled Jinhua ham [49]. Within the same quality grade of ham, BF showed higher ester content than SM, which is consistent with the results of a study on different muscles in smoked dry-cured ham [31]. Methyl isovalerate, ethyl isovalerate, and methyl pentanoate were identified as important odour-active esters in spoiled ham [49]. Similarly, in this study, ethyl isovalerate and methyl pentanoate were found to be higher in the BF of spoiled ham, while ethyl isovalerate was more abundant in the SM of spoiled ham.

Aldehydes play a crucial role in contributing to the overall flavour of dry-cured hams due to their low odour threshold [75]. Straight-chain aldehydes are mainly generated through autoxidation and oxidative deamination-decarboxylation of unsaturated fatty acids, while branched-chain aldehydes are predominantly produced through strecker degradation of amino acids [76]. In this study, the total aldehyde content is higher in spoiled hams compared to normal hams, potentially due to increased lipid oxidation and higher TBARS values in spoiled hams. Furthermore, this difference in aldehyde content might be influenced by the salt content in the ham. Previous findings have shown that smoked dry-cured hams with higher salt content during later stages of ripening exhibit lower aldehyde content [77]. The total aldehyde content of normal ham from the same muscle was significantly lower (*p* < 0.05) than that of spoiled ham in this study, potentially attributed to the high salt content in normal ham and its inhibitory effect on lipid oxidation [15]. The most abundant straight-chain aldehydes identified in Xuanwei ham were hexanal, nonanal, octanal, and heptanal, which originated from the oxidation of unsaturated fatty acids [78]. High levels of hexanal can result in an unpleasant, pungent, and putrid odour in dry-cured hams, while nonanal and octanal can contribute to unpleasant odours such as fat, oil, rancid or fried during ham maturation [79,80]. The present study showed that spoiled hams exhibit a high hexanal content, implying that it may be the main factor contributing to the spoiled flavour of Xuanwei hams [40]. Furthermore, the aldehyde content of BF was higher than that of SM, possibly due to the more intense drying and ripening processes involved in SM compared to BF. During this process, aldehydes can be converted into other volatile or non-volatile compounds [81]. This finding aligns with a study on Slovenian dry-cured hams [5], demonstrating a significantly higher aldehyde content in BF compared to SM (*p* < 0.05).

Acids play a significant role in contributing to the unique flavour of dry-cured ham [82]. In this study, the total acid content was significantly higher in spoiled ham compared to normal ham (*p* < 0.05). Among them, hexanoic acid, isovaleric acid, and valeric acid were the main acids in spoiled ham, while only hexanoic acid was detected in normal ham. The higher acid content in spoiled ham can be attributed to the microbial fermentation of free amino acids through the Strecker reaction, resulting in the production of acids [40]. According to Wang et al. [83], the acids contributed to a sweaty, cheesy, putrid, and sour odour. Therefore, the flavour difference between normal ham and spoiled ham in Xuanwei ham may stem from the presence of acids. There was no significant difference in acid content between the SM and the BF of normal hams, whereas a significant difference was observed in acid content between the SM and the BF of spoiled hams (*p* < 0.05).

Ketones are formed through lipid autoxidation or microbial metabolism [84]. They contribute to the aroma of dry-cured hams and have a fatty flavour associated with cooked meat and blue cheese odours [67]. In this study, 3-hydroxy-2-butanone was the main ketone of spoiled ham and normal ham. The results are consistent with previous findings on Iberian dry-cured hams [85], where 3-hydroxy-2-butanone was found in higher amounts in spoiled hams compared to normal hams. However, it should be noted that in Jinhua hams, 3-hdroxy-2-butanone was shown not to be key in differentiating between normal and spoiled ham [16]. The total ketone content in the BF of normal ham was higher than that in spoiled ham, with most of the ketones originating from microbial metabolism, specifically carbohydrate fermentation, lipid and ethyl ester oxidation, and amino acid catabolism [86]. Additionally, it was found that the ketone content was higher in BF than in SM in hams of the same grade quality (*p* < 0.05). Similar results were reported in Slovenian dry-cured hams, which were the result of fatty acid oxidation [5].

Alkanes, aromatics, and terpenes are classified as volatile compounds with higher odour thresholds that do not significantly contribute to flavour [31]. Our findings indicated that alkanes, pyrazines, and terpenes were present in higher content in normal ham compared to spoiled ham (*p* < 0.05). Conversely, aromatic compounds were found at higher levels in spoiled ham (*p* < 0.05). The main aromatic compounds detected in spoiled ham were p-xylene and o-xylene, which could potentially originate from the pig feed in Iberian dry-cured hams [87]. It is worth mentioning that an increase in aromatic compounds during maturation has been observed in dry-cured meat products and media inoculated with microorganisms [85]. Moreover, the levels of these compounds varied among samples of dry-cured ham inoculated with different microorganisms obtained from different locations [88].

It was worth mentioning that trimethylamine occurred among other volatile compounds and was detected only in spoiled ham. Trimethylamine belonged to the group of biogenic amines, which were degradation products produced by bacteria in meat products [89]. Trimethylamine has a rancid, fishy, fishy odour that is unacceptable and easily detectable with a low odour threshold [90]. 

#### 3.7.2. Major Volatile Compounds Analysis

To further examine the difference in the contribution of volatile compounds to the overall flavour profile of normal Xuanwei ham and spoiled Xuanwei ham, the OAV of each compound was calculated using the content of each volatile compound and the odour threshold value (OTV) obtained from previous literature [49]. It is generally accepted that volatile compounds play a greater role in the overall flavour when OAV ≥ 1 [91]. A total of 26 major volatile compounds (Table 4), including 6 aldehydes, 4 alcohols, 1 ketone, 9 esters, 3 acids, and 3 other compounds, were identified as significantly impacting the overall flavour profile of Xuanwei ham based on OAV. Specifically, 3-methyl butanal and hexanal were the primary contributors to flavour in normal ham. According to Zhou et al. [92], 3-methyl butanal imparted a malty aroma, providing a unique flavour to buckwheat honey. It is worth noting that 3-(methylthio)propionaldehyde contributed to the flavour of normal ham. Previous research by Etschmann et al. [93] showed that 3-(methylthio)propionaldehyde exhibited a strong odour reminiscent of soup, meat, onions, and potatoes. In this study, trimethylamine, methanethiol, hexanal, valeric acid, and hexanoic acid were found to have a significant impact on the flavour composition of spoiled ham. Among them, trimethylamine, methanethiol, and valeric acid were exclusive to spoiled ham. Trimethylamine contributed to a putrid fish flavour and fishy smell [90], methanethiol yielded an objectionable flavour [94], and valeric acid imparted a malodourous odour to spoiled ham [95]. The presence of these volatile compounds, which produce unpleasant odours, maybe the key factor responsible for the critical flavour differences between normal Xuanwei ham and spoiled Xuanwei ham.

### 3.8. Microbial Diversity Analysis

#### 3.8.1. Bacterial Diversity Analysis

**Figure 3:**
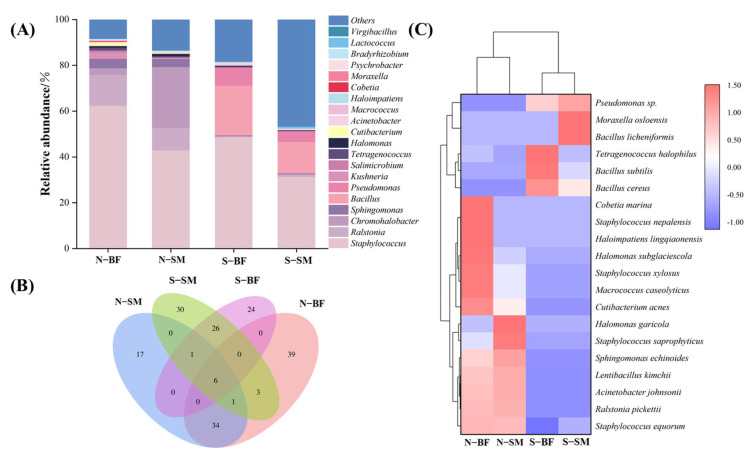
Bacterial genus level histograms (**A**), Wayne diagrams (**B**) and bacterial species level heat maps (**C**) of different muscles in normal and spoiled hams. N–SM: normal ham–semimembranosus; N−BF: normal ham−biceps femoris; S–SM: spoiled ham−semimembranosus; S–BF: spoiled ham–biceps femoris.

The single molecule real-time sequencing was utilised to analyse normal Xuanwei hams and spoiled Xuanwei hams at the bacterial genus and species levels, as well as to study the bacterial microbial community structure of Xuanwei hams with different quality grades and muscle types (Figure 3). At the genus level (Figure 3A), 142 bacterial genus were identified, with the top 20 dominant bacterial genus being *Staphylococcus*, *Ralstonia*, *Chromohalobacter*, *Sphingomonas*, *Bacillus*, *Pseudomonas*, *Kushneria*, *Salimicrobium*, *Tetragenococcus*, *Halomonas*, *Cutibacterium*, *Acinetobacter*, *Macrococcus*, *Haloimpatiens*, *Cobetia*, *Moraxella*, *Psychrobacter*, *Bradyrhizobium* and *Lactococcus*. In normal Xuanwei ham, *Staphylococcus* was the most abundant genus in both BF and SM, similar to Parma ham [11], followed by *Ralstonia* (13%) in the BF and *Chromohalobacter* (26%) in the SM. In contrast, *Bacillus* (39–41%) was the dominant genus in both BF and SM in spoiled hams, indicating a significant difference in the microbial community structure between normal and spoiled ham. It is worth mentioning that more *Lactococcus* were found in spoiled hams compared to normal hams (*p* < 0.05), consistent with a previous study by García et al. [21]. At the species level, the number of shared and unique OTUs between different samples was analysed, and a Venn diagram was plotted based on the results of OTU cluster analysis (Figure 3B). A total of 174 bacterial species were identified, with six species found in both the SM and BF of normal and spoiled hams. The clustered heat map at the species level (Figure 3C) revealed a significant difference between normal and spoiled hams, with *Staphylococcus equorum* being the dominant bacterial species in normal hams and differing significantly from spoiled hams. This finding is consistent with a previous study by Landeta et al. [24] on normal Spanish dry-cured hams. In spoiled hams, *Bacillus cereus*, *Bacillus subtilis,* and *Bacillus licheniformis* were the predominant species, and *Bacillus cereus* is known to be a significant cause of foodborne illness in humans [96], causing both gastrointestinal and non-gastrointestinal illnesses [97].

In healthy pigs, spoilage bacteria are not present inside the muscle. The entry of spoilage bacteria into the deeper layers of the muscle may occur in two ways. One is that the bacteria first grow on the surface of the leg and then penetrate internally; the other is that the bacteria enter the deeper layers of the muscle directly from the outside for various reasons [98]. One of the most likely ways that spoilage bacteria get into hams is through the leg meat getting contaminated when it is killed and split. These microorganisms get into the deeper part of the muscle through muscle wounds or through blood that does not drain from the blood vessels in time to reach the inner muscle [98]. Therefore, the main measures to prevent spoilage of dry-cured hams in industrialised production are to maintain environmental hygiene in the slaughtering and splitting of pigs, to drain the residual blood in the blood vessels of the carcasses, and to be careful, especially during the slaughtering of pigs.

#### 3.8.2. Fungal Diversity Analysis

**Figure 4:**
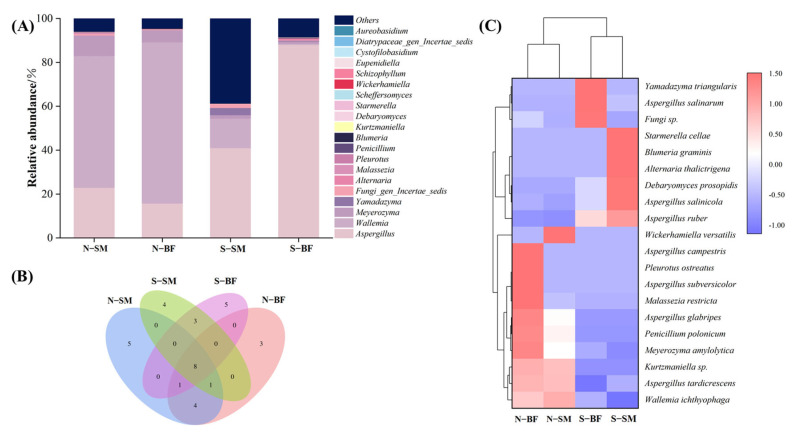
Fungal genus level histograms (**A**), Wayne diagrams (**B**) and fungal species level thermograms (**C**) of different muscles in normal and spoiled hams. N–SM: normal ham–semimembranosus; N–BF: normal ham−biceps femoris; S–SM: spoiled ham−semimembranosus; S–BF: spoiled ham–biceps femoris.

Figure 4A shows the top 20 dominant fungal genus in normal and spoiled hams: *Aspergillus*, *Wallemia*, *Meyerozyma*, *Yamadazyma*, *Fungi_gen_Incertae_sedis*, *Alternaria*, *Malassezia*, *Pleurotus*, *Penicillium*, *Blumeria*, *Kurtzmaniella*, *Debaryomyces*, *Starmerella*, *Scheffersomyces*, *Wickerhamiella*, *Schizophyllum*, *Eupenidiella*, *Cystofilobasidium*, *Diatrypaceae_gen_Incertae_sedis* and *Aureobasidium*. *Wallemia* was the most dominant fungal genus in normal hams, accounting for 60–73%, which is consistent with normal Nuodeng hams [99]. Meanwhile, *Aspergillus* was the most dominant fungal genus in spoiled hams, comprising 41–88%. It has been discovered that *Aspergillus* is the sole representative of the *Aspergillus* genus identified in spoiled ham; however, this species is typically not present in dry-cured ham. As a result, the presence of *Aspergillus* in spoiled ham may be attributed to contamination of the ham surface following contact with the surrounding environment [100]. From the Wayne diagram (Figure 4B), a total of 34 fungal species were identified, with eight species found in both the SM and BF in normal and spoiled hams. The clustered heat map at the species level (Figure 4C) revealed a significant difference distinguishing normal and spoiled hams. The most dominant fungal species in normal hams was *Wallemia ichthyophaga*, which was significantly and positively correlated with arginine, phenylalanine, leucine, isoleucine, and methionine in a study on Jinhua ham [25]. In contrast, *Aspergillus ruber* was the most dominant fungal species in spoiled hams. Interestingly, it is popular because it contains a substantial amount of secondary metabolites belonging to various classes, some of which show promising biological activities [101].

#### 3.8.3. LDA Analysis of Differential Microorganisms among Hams

**Figure 5:**
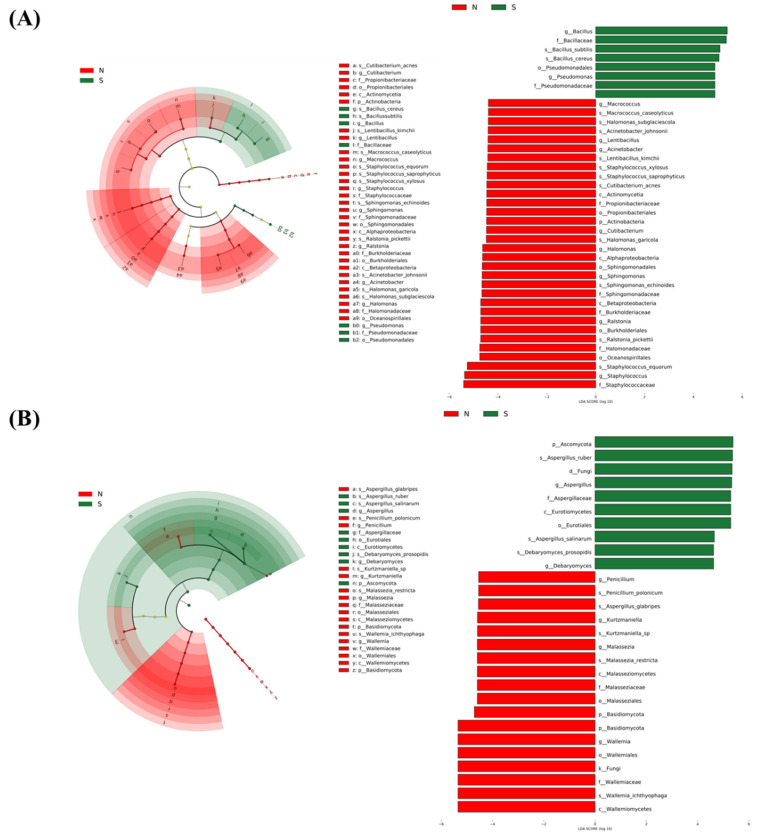
Linear discriminant analysis effect size (LEfSe) of the bacterial (**A**) and fungal (**B**) communities with an LDA score > 4.0 and *p* < 0.05. Cladograms indicate the phylogenetic distribution of microbial lineages among dry-cured hams. Circles represent phylogenetic levels from phylum to species. N: normal ham; S: spoiled ham.

LDA Effective Size (LEfSe) analysis enables not only comparisons between multiple groups but also subgroup comparative analyses within groups, thus realising species (i.e., biomarkers) with significant differences in abundance between groups [10]. Species with significant differences were ranked based on their LDA values, where higher values indicated a greater contribution to intergroup differences and greater importance as biomarkers [102]. Species with significant differences were ranked according to LDA values, and those with LDA > 4 were chosen for mapping. In Figure 5, the key microorganisms responsible for differences found in LEfSe between normal and spoiled hams were depicted, and the differential bacteria and fungi were more varied in normal hams than in spoiled hams. In the bacterial spectrum, *Staphylococcaceae*, *Alphaproteobacteria*, *Betaproteobacteria*, *Acinetobacter* and *Oceanospirillales* were the differential microorganisms in normal ham, while *Bacillaceaes* predominated in spoiled ham. In the fungal spectrum, *Basidiomycota* dominated in normal hams, while *Ascomycota* dominated in spoiled hams. Both normal hams and spoiled hams exhibited greater bacterial variation compared to fungi. Figure 5 demonstrates 16 species with LDA scores of 4 or higher, including 11 bacteria species and 5 fungi species. Among spoiled hams, 5 species had LDA scores exceeding 4, including 2 bacterial species and 3 fungal species. Normal hams displayed the highest variation in both bacterium and fungi. These findings confirm the diversity of bacterial and fungal species found in Xuanwei dry-cured hams and the variability between normal and spoiled hams.

### 3.9. Correlation Analysis between Microbial and Volatile Compounds

**Figure 6:**
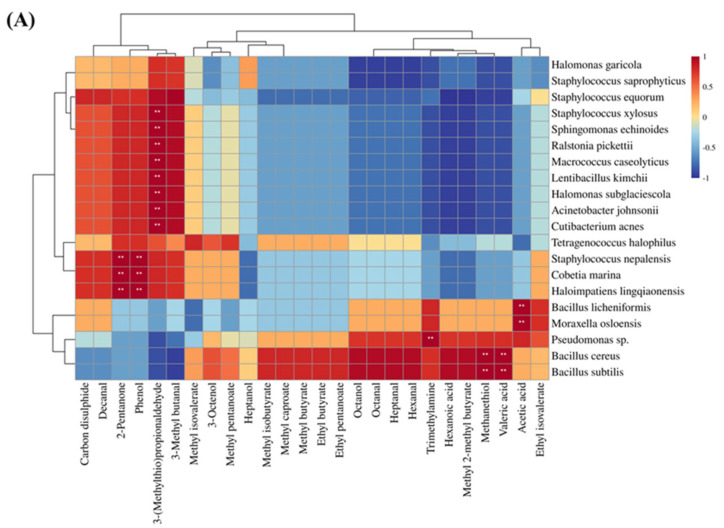

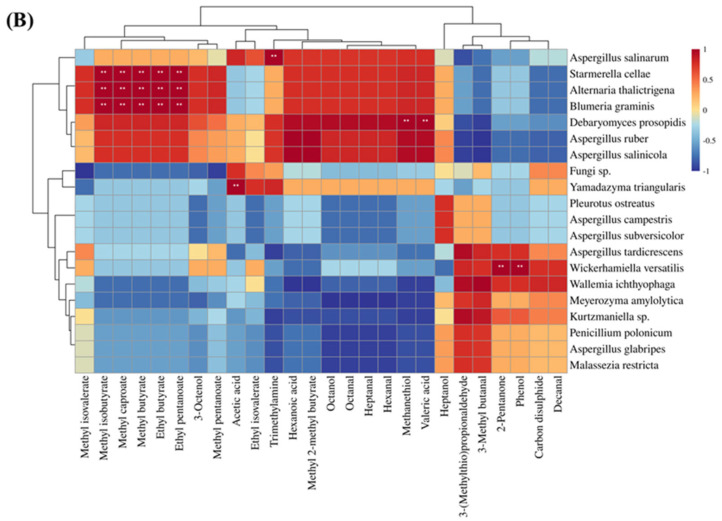
Heat map of bacterial (**A**) and fungal (**B**) species correlating with key flavour substances in normal and spoiled hams. **: extremely significant (*p* < 0.01).

The processing of dry-cured ham involves a series of biochemical and enzymatic reactions, including protein hydrolysis, lipid oxidation, and the Maillard reaction. These reactions are vital for the flavour formation of dry-cured ham, and the enzymatic activity of microorganisms also contributes to the production of flavour compounds [103]. To explore the relationship between off-odour in Xuanwei ham and microorganisms, a correlation analysis was conducted between key volatile compounds (OAV > 1) and microbial species. According to previous studies, microorganisms are the key factors affecting the flavour of different grades of ham in Jinhua [104]. The heatmap of the correlation between key volatile compounds and bacteria for both normal and spoiled ham is shown in Figure 6A. Trimethylamine, methanethiol, valeric acid, and hexanal were identified as the key volatile compounds that mainly contribute to the undesirable flavour in spoiled hams, particularly in the presence of *Bacillus cereus*, *Bacillus subtilis*, *Bacillus licheniformis*, *Pseudomonas sp.*, and *Moraxella osloensis* (*p* < 0.05). *Pseudomonas sp.* is an aerobic bacterium commonly associated with spoiled meat [22] and has also been found to have higher levels in spoiled hams than in normal hams in dry-cured hams [21]. *Moraxella osloensis* is a gram-negative aerobic bacterium known to be an opportunistic human pathogen responsible for diseases such as endocarditis, osteomyelitis, central venous catheter infections, and meningitis [105]. In summary, aerobic bacteria are more likely to cause spoiled ham in Xuanwei ham and also impact its flavour profile. Figure 6B illustrates the correlation thermograms between key volatile compounds of normal and spoiled ham with fungi. Spoiled ham was dominated by *Aspergillus ruber*, which correlated with most of the key volatile compounds, particularly exhibiting a strong positive correlation with trimethylamine. On the other hand, normal ham is dominated by *Wallemia ichthyophaga*, which has the highest positive correlation with 3-(methylthio)propionaldehyde and 3-methyl butanal. This suggested that *Wallemia ichthyophaga* promoted the production of 3-(methylthio)propionaldehyde and 3-methyl butanal, which impart favourable volatile compounds to Xuanwei normal ham. In conclusion, microorganisms play a vital role in determining the flavour profile of Xuanwei ham.

## 4. Conclusions

In conclusion, microorganisms play an important role in the formation of off-odour in Xuanwei hams. A total of 89 volatile compounds were identified in normal and spoiled hams, with 3-(methylthio)propionaldehyde and 3-methyl butanal being the major volatile compounds in normal hams. The levels of aldehydes and alcohols were significantly higher in spoiled hams compared to normal hams. The off-odour of spoiled hams was characterised by ichthyic, malodourous, sweaty, putrid, sour, and unpleasant odours, which were attributed to compounds such as trimethylamine, hexanal, octanal, methanethiol, and valeric acid. These compounds showed a positive correlation with *Bacillus cereus*, *Bacillus subtilis*, *Bacillus licheniformis*, *Pseudomonas* sp., *Aspergillus ruber*, and *Moraxella osloensis*. Additionally, high moisture, pH, and TBARS, as well as low NaCl content, were also responsible for the spoilage of hams with off-odour. This study provided valuable insight into the off-odour of Xuanwei ham from the perspective of microorganism communities. Further studies should focus on isolating and identifying the core spoiled strains responsible for off-odours in Xuanwei ham, as well as exploring methods for controlling off-odours in spoiled ham. These efforts will be beneficial in regulating spoilage during the production of Xuanwei hams, accelerating the industrialisation of Xuanwei ham, and maintaining consistent quality.

## Figures and Tables

**Table 1:** Physicochemical properties of different muscles in normal and spoiled hams.

Parameters	Normal Ham		Spoiled Ham	
	BF	SM	BF	SM
Moisture (g/100g)	46.88 ± 0.21 ^Ba^	39.44 ± 0.59 ^Bb^	56.32 ± 4.06 ^Aa^	49.98 ± 2.37 ^Ab^
a_w_	0.75 ± 0.00 ^Ba^	0.74 ± 0.00 ^Ba^	0.88 ± 0.00 ^Aa^	0.87 ± 0.00 ^Aa^
pH	5.74 ± 0.01 ^Bb^	5.79 ± 0.01 ^Ba^	6.63 ± 0.01 ^Ab^	6.45 ± 0.01 ^Aa^
NaCl (% of dry matter)	14.09 ± 2.10 ^Aa^	9.44 ± 0.68 ^Ab^	9.92 ± 1.12 ^Ba^	6.31 ± 0.88 ^Bb^
TBARS (mg MDA/kg)	0.31 ± 0.00 ^Bb^	0.46 ± 0.01 ^Ba^	1.84 ± 0.01 ^Ab^	1.98 ± 0.01 ^Aa^

Different uppercase letters (A,B) indicate significant differences between hams with different quality grades for the same muscle (*p* < 0.05), and different lowercase letters (a,b) indicate significant differences between different muscles for the ham with the same quality grade (*p* < 0.05). SM: Semimembranosus; BF: Biceps femoris; TBARS: lipid oxidation was assessed by thiobarbituric acid reactive substances; Results expressed as mean ± standard deviation (*n* = 6).

**Table 2:** Texture and colour parameters of different muscles in normal and spoiled hams.

Parameters	Normal Ham		Spoiled Ham	
	BF	SM	BF	SM
Texture				
Hardness (N)	148.80 ± 3.43 ^Ab^	242.01 ± 11.92 ^Aa^	71.76 ± 4.21 ^Bb^	78.52 ± 0.12 ^Ba^
Springiness (mm)	0.40 ± 0.03 ^Aa^	0.41 ± 0.05 ^Aa^	0.26 ± 0.05 ^Ba^	0.29 ± 0.02 ^Ba^
Cohesiveness	0.49 ± 0.01 ^Aa^	0.39 ± 0.06 ^Ab^	0.46 ± 0.01 ^Aa^	0.42 ± 0.02 ^Ab^
Chewiness (N × mm)	30.18 ± 3.55 ^Aa^	39.20 ± 13.14 ^Aa^	10.62 ± 1.44 ^Bb^	13.91 ± 1.15 ^Ba^
Resilience	0.19 ± 0.02 ^Aa^	0.18 ± 0.03 ^Aa^	0.19 ± 0.01 ^Aa^	0.17 ± 0.02 ^Aa^
Colour				
L*	43.26 ± 0.06 ^Ba^	37.56 ± 0.08 ^Bb^	47.09 ± 0.03 ^Aa^	41.62 ± 0.09 ^Ab^
a*	19.56 ± 0.16 ^Ba^	16.32 ± 0.32 ^Bb^	23.06 ± 0.07 ^Aa^	20.15 ± 0.19 ^Ab^
b*	15.82 ± 0.35 ^Ba^	12.33 ± 0.12 ^Bb^	19.32 ± 0.62 ^Aa^	15.73 ± 0.39 ^Ab^

Different uppercase letters (A,B) indicate significant differences between hams with different quality grades for the same muscle (*p* < 0.05), and different lowercase letters (a,b) indicate significant differences between different muscles for the ham with the same quality grade (*p* < 0.05). SM: Semimembranosus; BF: Biceps femoris. L*: lightness, a*: redness, b*: yellowness. Results are expressed as mean ± standard deviation (*n* = 6).

**Table 3:** Volatile compounds (μg/kg) of different muscles in normal and spoiled hams.

Volatile Compounds		RI	Normal Ham		Spoiled Ham	
			BF	SM	BF	SM
Alcohols						
1	Methanethiol	672	ND	ND	12.85 ± 1.01 ^a^	5.99 ± 0.60 ^b^
2	Isopropyl alcohol	928	ND	0.39 ± 0.09	ND	ND
3	Ethanol	934	105.84 ± 35.04 ^Aa^	46.96 ± 4.61 ^Bb^	106.28 ± 25.72 ^Ab^	170 ± 29.96 ^Aa^
4	2-Butanol	1030	3.35 ± 3.39	ND	ND	ND
5	3-Pentanol	1117	0.51 ± 0.13 ^B^	ND	2.28 ± 0.8 ^Aa^	1.28 ± 0.24 ^a^
6	2-Pentanol	1129	9.75 ± 3.59 ^Aa^	1.53 ± 0.20 ^Bb^	3.6 ± 0.2 ^Ba^	2.48 ± 0.48 ^Ab^
7	Butanol	1151	8.94 ± 2.02 ^Aa^	1.63 ± 0.32 ^Ab^	2.24 ± 0.64 ^Ba^	2.2 ± 0.56 ^Aa^
8	1,3-Pentenol	1165	10.12 ± 3.58 ^Aa^	4.53 ± 0.50 ^Aa^	10.56 ± 2.8 ^Aa^	6 ± 1.64 ^Aa^
9	Isoamyl alcohol	1212	6.13 ± 1.88 ^Ba^	7.75 ± 0.38 ^Ba^	47.92 ± 4.24 ^Aa^	22.68 ± 3.64 ^Ab^
10	3-Methyl butenol	1251	ND	1.63 ± 0.39	ND	ND
11	Pentanol	1255	99.00 ± 30.43 ^Aa^	6.55 ± 0.31 ^Bb^	47.28 ± 12.2 ^Aa^	24.16 ± 4.6 ^Ab^
12	2-Ethyl butanol	1305	2.69 ± 0.86	ND	ND	ND
13	Hexanol	1356	ND	ND	123.76 ± 11 ^a^	75.28 ± 16.32 ^b^
14	3-Octenol	1456	31.83 ± 3.12 ^Ba^	8.69 ± 3.27 ^Bb^	36.4 ± 4.88 ^Aa^	29.52 ± 6 ^Aa^
15	Heptanol	1460	6.50 ± 2.93 ^Aa^	7.56 ± 4.29 ^Aa^	7.16 ± 1.48 ^Aa^	6.96 ± 1.36 ^Aa^
16	Benzyl alcohol	1508	ND	ND	1.76 ± 0.52	ND
17	Octanol	1564	7.07 ± 1.58 ^Ba^	4.37 ± 1.17 ^Ba^	20.52 ± 3.32 ^Aa^	19.2 ± 4.32 ^Aa^
18	2,3-Butanediol	1580	24.66 ± 3.23 ^Ab^	45.42 ± 3.65 ^Aa^	11.6 ± 2.2 ^Ba^	9.96 ± 2.28 ^Ba^
19	Trans-2-Octenol	1620	9.29 ± 3.70 ^A^	ND	3.48 ± 0.44 ^Aa^	2.56 ± 0.44 ^a^
20	2-Phenylethanol	1912	ND	0.79 ± 0.31 ^B^	5.16 ± 1.32 ^a^	4.4 ± 1.2 ^Aa^
21	Dodecanol	1999	ND	ND	1.88 ± 0.72 ^a^	3.6 ± 1.48 ^a^
	Total		325.65 ± 95.48 ^Aa^	137.82 ± 18.98 ^Bb^	444.73 ± 72.49 ^Aa^	386.27 ± 75.12 ^Aa^
Esters						
22	Methyl propionate	905	ND	ND	2.65 ± 0.85	ND
23	Methyl isobutyrate	920	ND	1.18 ± 0.23	3.60 ± 0.93	ND
24	Ethyl propionate	949	1.2 ± 0.12	ND	ND	ND
25	Isobutyl acetate	960	1.64 ± 0.32	ND	ND	2.6 ± 0.28
26	Methyl butyrate	983	1.44 ± 0.16 ^Bb^	6.27 ± 1.27 ^Aa^	14.67 ± 3.35 ^Aa^	1.2 ± 0.48 ^Bb^
27	Methyl 2-methyl butyrate	1009	2.08 ± 0.12 ^Ba^	2.76 ± 0.59 ^Aa^	8.65 ± 0.34 ^Aa^	3.48 ± 0.56 ^Ab^
28	Methyl isovalerate	1018	6.96 ± 0.4 ^Aa^	4.59 ± 0.96 ^b^	14.47 ± 0.80 ^B^	ND
29	Ethyl butyrate	1036	ND	ND	7.02 ± 0.28	ND
30	Ethyl 2-methyl butyrate	1053	4.12 ± 0.4 ^Aa^	0.32 ± 0.04 ^Bb^	3.46 ± 0.85 ^Ab^	6.28 ± 0.92 ^Aa^
31	Ethyl isovalerate	1070	7 ± 0.32 ^Aa^	ND	6.09 ± 0.88 ^Ab^	10.8 ± 1 ^a^
32	Methyl pentanoate	1089	2.96 ± 0.4 ^Ba^	1.35 ± 0.51 ^b^	19.86 ± 0.88 ^A^	ND
33	Ethyl pentanoate	1137	ND	ND	7.24 ± 4.49	ND
34	Methyl caproate	1187	8.72 ± 1.2 ^Aa^	7.10 ± 0.87 ^Aa^	10.26 ± 0.18 ^Aa^	4.69 ± 0.48 ^Bb^
35	Ethyl caproate	1234	8.2 ± 1 ^Aa^	1.93 ± 0.53 ^Ab^	17.57 ± 2.20 ^Ba^	16.48 ± 2.2 ^Ba^
36	Ethyl caprylate	1434	2.12 ± 0.72 ^A^	ND	1.43 ± 0.02 ^Ab^	3.76 ± 0.16 ^a^
37	Gamma-caprolactone	1687	6.8 ± 0.56 ^Ba^	1.24 ± 0.30 ^Bb^	14.48 ± 3.07 ^Ab^	7.08 ± 0.92 A^a^
38	γ-Oenantholacton	1789	ND	ND	3.10 ± 0.52	ND
39	Gamma-octalactone	1903	4.32 ± 0.64 ^A^	ND	2.78 ± 0.87 ^Bb^	5.44 ± 0.6 ^a^
40	Gamma-nonanoic lactone	2018	ND	0.72 ± 0.19	ND	ND
	Total		57.56 ± 6.36 ^Ba^	27.45 ± 5.50 ^Bb^	137.32 ± 20.50 ^Aa^	61.81 ± 7.6 ^Ab^
Aldehydes						
41	Butyraldehyde	900	9.95 ± 0.34 ^a^	5.27 ± 0.57 ^b^	ND	ND
42	2-Methyl butanal	910	22.02 ± 0.71 ^Aa^	15.24 ± 0.16 ^Ab^	10.36 ± 0.28 ^Ba^	8.96 ± 0.48 ^Bb^
43	3-Methyl butanal	913	26.66 ± 0.65 ^Aa^	18.46 ± 0.40 ^Ab^	12.32 ± 0.96 ^Ba^	13.16 ± 0.56 ^Ba^
44	Pentanal	958	ND	ND	8.28 ± 2.68 a	5.16 ± 1 a
45	Hexanal	1082	81.34 ± 8.08 ^Ba^	22.66 ± 0.74 ^Bb^	206.46 ± 5.32 ^Aa^	136.72 ± 10.08 ^Ab^
46	Heptanal	1182	13.81 ± 0.77 ^Ba^	8.18 ± 1.52 ^Bb^	43.28 ± 2.56 ^Aa^	36.24 ± 0.6 ^Aa^
47	Octanal	1286	20.61 ± 0.32 ^Ba^	15.35 ± 0.84 ^Bb^	59.52 ± 4.88 ^Aa^	52.52 ± 2.76 ^Aa^
48	(E)-2-Heptenal	1318	6.82 ± 2.02	ND	ND	ND
49	Nonanal	1390	36.74 ± 0.74 ^Bb^	49.75 ± 0.99 ^Ba^	143.04 ± 7.88 ^Aa^	175.96 ± 20.36 ^Aa^
50	(E)-2-Octenal	1422	1.26 ± 0.55	ND	ND	ND
51	3-(Methylthio)propionaldehyde	1446	7.30 ± 2.28 ^a^	2.23 ± 1.23 ^b^	ND	ND
52	Decanal	1495	2.37 ± 0.47 ^a^	1.83 ± 0.13 ^Aa^	ND	1.92 ± 0.68 ^A^
53	Benzaldehyde	1511	19.89 ± 0.91 ^Aa^	10.76 ± 0.77 ^Ab^	10.4 ± 0.6 ^Ba^	7.24 ± 0.2 ^Bb^
54	Phenylacetaldehyde	1631	12.53 ± 0.80 ^Ab^	14.97 ± 0.75 ^Aa^	4.68 ± 0.64 ^Ba^	4.96 ± 0.92 ^Ba^
55	3-Heptylacrolein	1637	2.25 ± 1.16	ND	ND	ND
	Total		263.57 ± 15.79 ^Ba^	164.7 ± 8.09 ^Bb^	498.34 ± 25.8 ^Aa^	420.72 ± 37.64 ^Ab^
Acids						
56	Acetic acid	1463	ND	ND	ND	8.19 ± 3.62
57	Butyric acid	1659	ND	ND	ND	3.99 ± 0.59
58	Isovaleric acid	1688	ND	ND	20.99 ± 5.87 ^a^	15.77 ± 0.18 ^a^
59	Valeric acid	1767	ND	ND	15.08 ± 4.51 ^a^	7.21 ± 1.44 ^b^
60	Hexanoic acid	1880	4.12 ± 1.88 ^Ba^	4.24 ± 1.48 ^Ba^	93.68 ± 5.05 ^Aa^	66.53 ± 10.36 ^Ab^
61	Heptanoic acid	1986	ND	ND	ND	2.7 ± 0.81
62	Octanoic acid	2107	ND	ND	6.11 ± 0.49 ^b^	9.35 ± 1.21 ^a^
63	Capric acid	2327	ND	ND	5.27 ± 1.71 ^b^	11.58 ± 0.89 ^a^
64	Myristic acid	2717	ND	ND	16.17 ± 3.25 ^a^	13.98 ± 4.88 ^a^
	Total		4.12 ± 1.88 ^Ba^	4.24 ± 1.48 ^Ba^	157.31 ± 20.87 ^Aa^	139.31 ± 23.98 ^Ab^
Ketones						
65	Acetone	806	11.22 ± 7.91 ^a^	6.56 ± 0.91 ^a^	ND	ND
66	2-Pentanone	974	49.62 ± 7.02 ^a^	13.02 ± 2.52 ^b^	ND	ND
67	4-Methyl-3-hexanone	1074	ND	0.78 ± 0.37	ND	ND
68	2-Heptanone	1176	ND	ND	25.72 ± 4.2 ^a^	16.84 ± 2.8 ^a^
69	3-Hydroxy-2-butanone	1281	7.16 ± 0.17 B^a^	5.77 ± 0.38 ^Ab^	10.68 ± 0.12 ^Aa^	6.96 ± 0.76 ^Ab^
70	Methyl hepten	1335	10.16 ± 0.69 ^Aa^	3.58 ± 0.21 ^Ab^	4.48 ± 0.56 ^Ba^	4.24 ± 0.8 ^Aa^
71	Hydroxy-2-butanone	1365	0.91 ± 0.15	ND	ND	ND
	Total		79.06 ± 15.93 ^Aa^	29.7 ± 4.39 ^Ab^	40.88 ± 4.88 ^Ba^	28.04 ± 4.36 ^Ab^
Alkanes						
72	3-Methylnonane	961	4.53 ± 2.41	ND	ND	ND
73	Isododecane	1157	ND	ND	ND	1.72 ± 0.32
74	Hexadecanal	2133	7.89 ± 2.65 ^a^	5.58 ± 3.31 ^a^	ND	ND
	Total		12.42 ± 5.07 ^a^	5.58 ± 3.31 ^Aa^	ND	1.72 ± 0.32 ^B^
Pyrazines						
75	2-Methylpyrazine	1263	3.04 ± 0.72 ^a^	1.88 ± 0.36 ^b^	ND	ND
76	2,6-Dimethylpyrazine	1325	8.52 ± 0.04 ^a^	5.08 ± 7.4 ^b^	ND	ND
77	2-Ehtyl-6-methylpirazine	1381	1.4 ± 0.2 ^a^	ND	ND	1.17 ± 0.64 ^a^
78	2,3,5-Trimethylpyrazine	1402	19.96 ± 1.36 ^a^	12.4 ± 2.36 ^b^	ND	1.81 ± 0.73 ^c^
	Total		32.92 ± 2.32 ^a^	19.36 ± 10.12 ^Ab^	ND	2.97 ± 1.35 ^B^
Aromatic						
79	Ethylbenzene	1121	1.76 ± 0.00 ^B^	ND	3.56 ± 0.76 ^Aa^	3.28 ± 0.72 ^a^
80	P-Xylene	1137	ND	1.39 ± 0.62 ^a^	4.8 ± 1.28 ^Ab^	3.64 ± 1.16 ^B^
81	O-Xylene	1176	1.56 ± 0.10 ^Aa^	0.68 ± 0.29 ^Aa^	8.92 ± 0.68 ^Bb^	8.48 ± 2 ^Ba^
82	Phenol	2008	6.76 ± 0.48 ^a^	3.8 ± 1.28 ^b^	ND	ND
	Total		3.32 ± 0.10 ^Ba^	2.07 ± 0.91 ^Ba^	24.04 ± 3.2 ^Aa^	19.2 ± 5.16 ^Ab^
Terpenes						
83	Cyclooctatetraene	1316	8.44 ± 0.92	ND	ND	ND
84	Longifolene	1378	6.64 ± 0.96 ^a^	7.56 ± 1.32 ^a^	ND	ND
85	Trans-2-Octenol	1399	3.48 ± 0.44 ^a^	2.56 ± 0.44 ^a^	ND	ND
	Total		18.92 ± 2.32 ^a^	10.12 ± 1.76 ^b^	ND	ND
Others						
86	Trimethylamine	633	ND	ND	5.09 ± 3.62 ^b^	13.05 ± 1.61 ^a^
87	Carbon disulphide	713	9.63 ± 0.66 ^Aa^	2.73 ± 0.37 ^Bb^	1.88 ± 0.28 ^Bb^	4.92 ± 0.08 ^Aa^
88	2-Pentylfuran	1230	1.10 ± 0.27 ^Ba^	0.75 ± 0.28 ^Ba^	3.64 ± 0.84 ^Aa^	2.64 ± 0.2 ^Aa^
89	Hexanenitrile	1293	5.29 ± 1.46 ^a^	2.30 ± 0.55 ^b^	ND	ND
	Total		16.01 ± 2.38 ^Aa^	5.77 ± 1.20 ^Bb^	10.61 ± 4.74 ^Bb^	20.61 ± 1.69 ^Aa^

Different uppercase letters (A,B) indicate significant differences between hams with different quality grades for the same muscle (*p* < 0.05), and different lowercase letters (a,b) indicate significant differences between different muscles for the ham with the same quality grade (*p* < 0.05). ND: Compounds were not detected in GC–MS. SM: Semimembranosus. BF: Biceps femoris. RI—retention indices. Results are expressed as mean ± standard deviation (*n* = 6).

**Table 4:** Key volatile compounds of different muscles in normal and spoiled hams (OAV > 1).

	Volatile Compounds	OTV (µg/kg)	Normal Ham		Spoiled Ham	
			BF	SM	BF	SM
1	Trimethylamine	0.032	0	0	159.06	407.81
2	Methanethiol	0.07	0	0	183.61	85.57
3	Methyl 2-methyl butyrate	1	2.08	2.76	8.65	3.48
4	3-Methyl butanal	0.1	266.64	184.59	123.2	131.6
5	Methyl isobutyrate	1.9	0	0	1.9	0
6	2-Pentanone	28	1.77	0	0	0
7	Methyl butyrate	7.1	0	0	2.07	0
8	Methyl isovalerate	2.2	3.16	2.08	6.58	0
9	Ethyl butyrate	0.04	0	0	175.6	0
10	Ethyl isovalerate	3	2.33	0	2.03	3.6
11	Hexanal	0.28	290.5	80.93	737.36	488.28
12	Methyl pentanoate	2.2	1.34	0	9.02	0
13	Ethyl pentanoate	0.11	0	0	65.82	0
14	Heptanal	3	4.6	2.73	14.43	12.08
15	Methyl caproate	10	0	0	1.26	0
16	Octanal	0.7	29.44	21.93	85.03	75.03
17	Carbon disulphide	0.34	87.15	8.03	5.53	14.92
18	3-(Methylthio)propionaldehyde	0.63	11.59	3.53	0	0
19	3-Octenol	2	15.91	4.35	18.2	14.76
20	Heptanol	4.8	1.35	1.57	1.49	1.45
21	Acetic acid	6	0	0	0	1.09
22	Decanal	0.4	5.93	4.58	0	4.8
23	Octanol	2.7	2.52	1.56	7.33	6.86
24	Valeric acid	0.037	0	0	412.97	194.86
25	Hexanoic acid	0.6	6.87	7.07	156.13	110.88
26	Phenol	5.6	1.23	0	0	0

Odour threshold value (OTV) and odour threshold value (OAV) represented odour threshold value and odour active value, respectively. SM: Semimembranosus; BF: Biceps femoris.

## Data Availability

The original contributions presented in the study are included in the article, further inquiries can be directed to the corresponding author.
